# Atraumatic femoral neck fracture during bisphosphonate treatment: case report and review of the literature

**DOI:** 10.1007/s40520-017-0846-0

**Published:** 2017-10-27

**Authors:** Robert Wilk, Damian Kusz, Hanna Grygiel, Magdalena Grosiak, Jakub Kamiński, Marcin Kusz

**Affiliations:** 1 Department of Orthopaedics and Traumatology, School of Medicine in Katowice, Medical University of Silesia in Katowice, Ziołowa 45/47 Street, 40-635 Katowice, Poland; 2 Department of Orthopaedics and Traumatology, Students’ Scientific Society, School of Medicine in Katowice, Medical University of Silesia in Katowice, Katowice, Poland

## Introduction

Postmenopausal osteoporosis is a metabolic bone disease that affects a significant part of the population. Preventing fractures of the femoral neck is one of the most pressing issues in the treatment of osteoporosis. Bisphosphonates (BPs) are highly recommended in people suffering from osteoporosis. They are proven to inhibit bone resorption and also proven to have anti-fracture efficiency. However, treatment using BPs does not completely eliminate the risk of fractures. In this article we present a female patient with a non-traumatic fracture of the femoral neck who regularly ingested oral BPs for 3 years as a preventative treatment.

## Case study

**Fig. 1:**
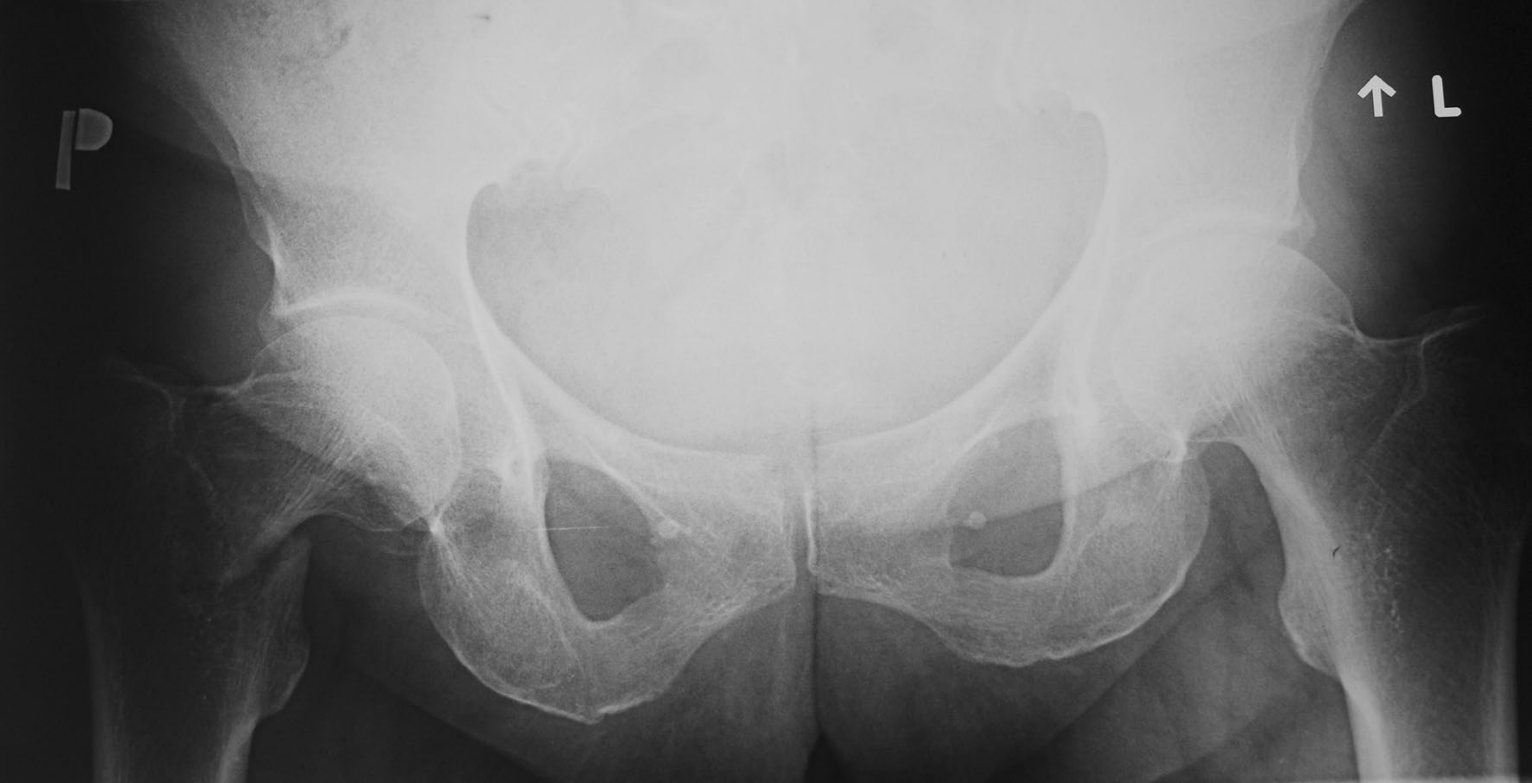
The anteroposterior pelvis radiograph with a basicervical fracture of the proximal right femur

**Fig. 2:**
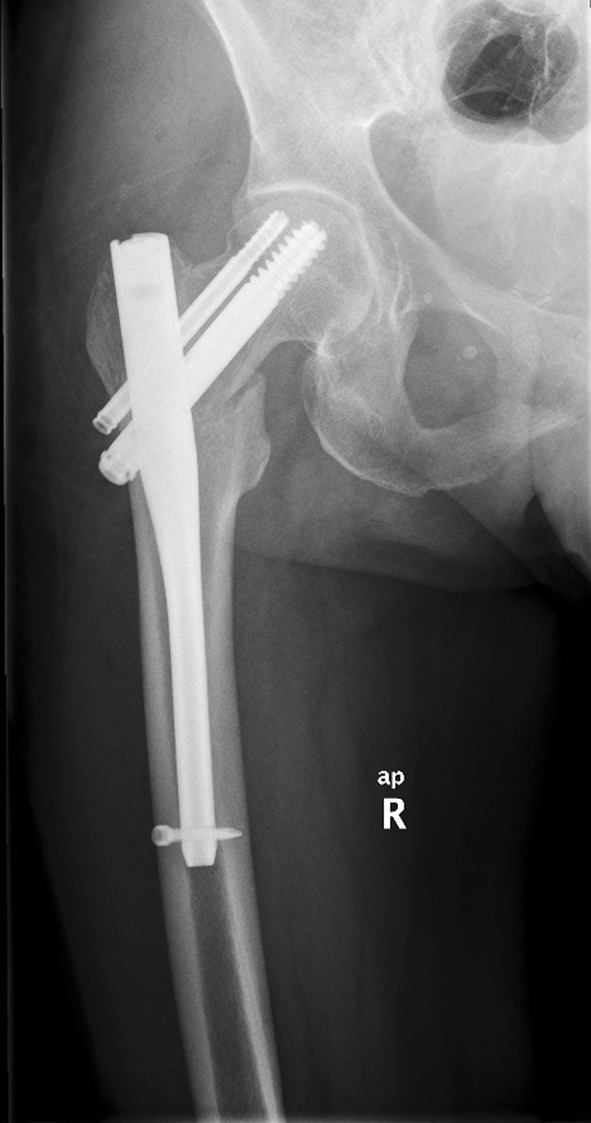
The anteroposterior radiograph after the surgery

**Fig. 3:**
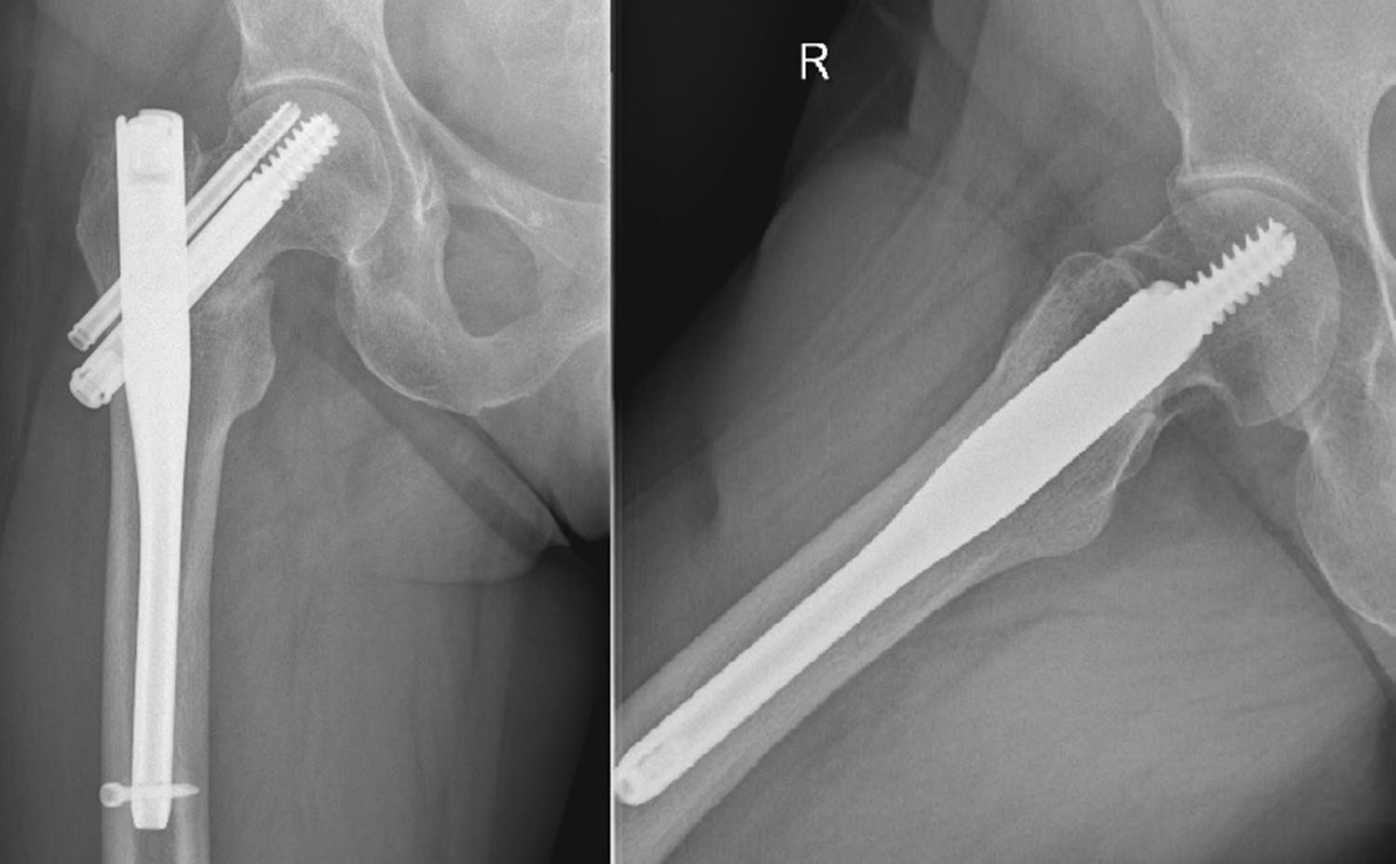
The anteroposterior and axial radiograph 3 months after the surgery

A 57-year-old woman was admitted to the Department of Orthopaedics and Traumatology because of severe pain localised on the lateral side of the right thigh and the right groin. The patient complained of pain a month prior to admission. There was no known history of injury due to falling. Despite the pain, the patient was able to walk unassisted. Then after 1 week the patient lifted minor weights which caused a slight hip sprain during flexion of the spine, the patient’s condition rapidly deteriorated. Initially, lumbar radiculopathy was misdiagnosed. The patient was treated with nonsteroidal anti-inflammatory drugs, specifically dexketoprofen, naproxen and ketoprofen, first orally and secondly intramuscular. The treatment was ineffective and the pain increased; subsequently the patient was taken to the Accident and Emergency Department. During initial clinical examination shortening and external rotation of the right lower limb was revealed. Lasègue test was negative. Due to intense pain the patient’s mobility was limited and she was unable to bear any weight; crutches were given to assist with walking. Her past medical history revealed osteoporosis, no other complication was indicated. Due to an ankle sprain 3 years prior a dual-energy X-ray absorptiometry (DEXA) was performed by a general practitioner which revealed low bone density. The patient had a *T* score of − 2.7 and *Z* score of − 1.7 at the lumbar spine. The patient was treated with an oral BP (ibandronic acid 150 mg monthly), plus an oral supplementation of vitamin D3 (1250 U daily) or vitamin D3 plus calcium (1000 U and 500 mg daily, respectively) for 3 years, alternately. The patient’s occupation was a caretaker at a nursery school for 28 years. She describes her job as ‘slightly physical’. She reached menopause 8 years ago, no hormone replacement therapy was used. There was no known history of smoking or alcohol abuse. Her physical activity was normal and she did not reveal any eating disorders. Her mother also suffered from osteoporosis. Analysis of the blood prior to the surgery revealed low levels of sodium chloride and creatinine, and a high platelet count. All other laboratory tests were within normal range (Table 1). According to the standard protocol the levels of Vitamin D and parathormone serum were not checked. She did not present any clinical symptoms of hyperparathyroidism. The patient’s height was 159 cm, weight was 56 kg, and Body Mass Index (BMI) was 22.15. Radiographs taken prior to admission revealed a basicervical fracture of the proximal right femur with slight displacement (vertical, smooth-looking fracture line). There were no other pathological signs (Fig. 1). The neck-shaft angle on the contralateral proximal femur was 130°. The chest x-ray did not reveal any pathology besides slight atherosclerosis of the aorta and degenerative changes of the thoracic spine. She underwent surgery for closed reduction and internal fixation of fracture using titanium trochanteric nail (130°/10 × 180 mm) (Fig. 2). Post-surgical treatment included physical therapy and the patient continued pre-fractural osteoporosis treatment which included Ibandronic acid. Ibandronic acid was not converted into other drugs such as Teriparatide. Normal fracture healing was observed 3 months post-surgery (Fig. 3). A subsequent follow-up was conducted 8 months post-surgery which revealed the patient walked without crutches. However, the patient limped and complained of slight pain at the right hip.

**Table 1:** Blood tests at admission

Test	Patient’s result	Normal range
White blood cells (x10^3^/μl)	8.09	4–10
Red blood cells (x10^6^/μl)	4.32	4–5
Heamoglobin (g/dl)	12.9	12–16
Hematocrit (vol%)	36.7	36–47
Platelets (x10^3^/μl)	**353**	135–350
Total protein (g/dl)	7.13	6.4–8.3
Total bilirubin (mg/dl)	0.17	0-1.2
Creatinin (mg/dl)	**0.4**	0.51–0.95
Sodium (mmol/l)	**133**	136–142
Chloride (mmol/l)	**92**	97–107
Potassium (mmol/l)	3.5	3.4–5.1

## Discussion

Nowadays, BPs are the first-line treatment for osteoporosis worldwide [1]. However, they have some possible side effects, such as atypical fractures [2, 3]. There are a wide variety of fractures reported at rare locations such as the distal fibula, ulna, tibia, metatarsus, pubis, ilium; however, fractures of the femur (subtrochanteric or diaphyseal) are the most common [4–10]. Our patient presented a basicervical fracture which is new and at a very unexpected location. Several cases of such fractures were reported to date. Previous reports described patients treated with different BPs—alendronic acid (three patients), cyclical etidronate (one patient) and one with ibandronic acid similar to the patient in this study [11–14]. Although treatment with BPs is highly recommended and commonly prescribed for osteoporosis to reduce the risk of hip fracture, preceding case reports show that it may also provoke femoral neck fracture.

According to the available data, ibandronic acid has vertebral and non-vertebral anti-fracture efficiency, especially in women with postmenopausal osteoporosis. It activity causes dysfunction as well as apoptosis in osteoclasts and reduces bone turnover consequently. In postmenopausal women, treatment using BPs reduces the elevated rate of bone turnover, leading to, on average, a net gain in bone mass. However, extended over-suppression of bone turnover and elasticity is considered to be vital in the atypical fractures [15].

Atraumatic fractures of different localisation are commonly described as stress fractures. Femoral neck stress fractures can be divided into two types: fatigue and insufficiency. Fatigue type fractures are distinctive for athletes (especially in long-distance runners) and military staff [16–18]. They are the result of abnormal stress loaded onto a healthy bone. The second (insufficiency) type is caused by the impairment of normal muscular force applied to poorly structured bone with lowered elasticity. This type is more common in elderly patients, and patients suffering from postmenopausal osteoporosis as well as other bone affecting diseases (e.g., osteomalacia, hyperparathyroidism, osteitis fibrosa, Paget’s disease). Moreover, there are rare reports of insufficiency fractures association with rheumatoid arthritis, diabetes mellitus, chronic renal failure, epilepsy, transient osteopenia during pregnancy, cystic fibrosis, use of steroids, carbamazepine, or pelvic irradiation [19–26]. Stress fracture risk may be also escalated by geometrical parameters of the bone, such as lateral bowing of femur and small neck-shaft angle [10, 19]. The patient in this study presented a fracture caused by normal loading of an abnormal, osteoporotic bone with no comorbidities or medication usage apart from the intake of BPs.

According to the guidelines set by the American Society of Bone and Mineral Research (ASBMR) atraumatic fractures during therapy using BPs are defined as atypical fractures. According to this description atypical fractures could not be diagnosed over the lesser trochanter (fracture of the femoral neck is an excluding feature) [27]. Therefore, according to these conditions our case is not a normal atypical fracture.

The possible fracture mechanism is related to osteoporosis (worse bone stock) and accumulation of microdamage compounded by suppressed bone turnover due to BPs use (akin to atypical fractures) [27]. The risk of such fractures seems to be associated with the duration of BPs therapy. There are no precise guidelines on how long therapy should be administered. BPs are chemically stable, seldom metabolised and have a half-life of over 10 years in the skeletal system [14]. Therefore, both positive and negative effects may persist after discontinuation of the therapy. According to ASBMR, treatment for 5 years or more is associated with an increased risk of atypical (subtrochanteric or femoral shaft) fracture. Our patient presented basicervical fracture after 3 years of BP treatment, which is categorised as an intermediate duration [28]. It is also important to note that the molecular and clinical effect of various BPs is not equal. For example, the lower binding affinity of ibandronate with the mineral component of bone in comparison to zoledronate or alendronate might potentially be associated with lower risk of atypical fractures [26]. Ibandronate is regarded as one of the safest and most effective BP, nevertheless the patient in this study as well as one other patient from a different study developed a fracture during treatment with ibandronate [14].

All of the non-traumatic femoral neck fractures associated with BPs treatment were only described in women aged over 77 [11–14]. This is because BPs are mostly prescribed in older patients (our patient was only 57). In other cases the *T* score was not always less than − 2.5. Prodromal pain was present in all cases in the groin area without any histories of falling. In the anamnesis, there was no evidence of excessive physical activity (in relation to age) [11–14]. Most patient had no comorbidities, routine blood tests were within normal range as well as levels of vitamin D and parathyroid hormone [11–14]. Only one of the patients presented hyperparathyroidism and mildly elevated creatinine level (however she had normal serum estimations of serum calcium and vitamin D) [11]. In our case vitamin D, calcium and parathormone serum levels were not checked (this is the limitation of this case report). The weight and height was given in only one case [12]. Similar to our patient, the other patient had a petite physique (height 150 cm, body weight 43 kg, BMI 19.1 kg/m2). Therefore, it seems that insufficiency hip fractures could be a consequence of extremely high doses of BPs associated with long half-life (nowadays BP`s are not administered according to the weight and level of osteoporosis) and extended over-suppression of bone turnover and lower elasticity [12]. In other cases fractures appeared 7–10 years after taking alendronic acid and 4 years after taking ibandronate [11, 13, 14]. In one case the fracture was diagnosed 3 months after treatment was started [the patient was treated with oral cyclical etidronate (400 mg/day for 2 weeks every 3 months)] [12].

Though all BP’s are considered to be safe, the possible side effects including atraumatic fractures should be always kept in mind. Diagnostic tools (X-ray, CT, MRI, and scintigraphy) should be performed as soon as possible, especially if a patient reports pain in the groin during BPs’ treatment. Early diagnosis can be difficult—about 75% of femoral neck stress fractures are either missed or misdiagnosed during initial examination [18]. The symptoms are non-specific and radiographs do not always reveal characteristic fractural features (unlike subtrochanteric and femoral shaft fractures). Delay in diagnosis and treatment (conservative or surgical) can lead to serious complications associated with secondary displacement. Avascular necrosis of the femoral head, non-union, or various deformities occur in 30% of such cases [18]. In most cases it is necessary to replace the broken bone with prosthesis. On the other hand, quick diagnosis of non-displaced fractures can be successfully treated conservatively (non-surgically) with absolute bed rest > 4 weeks.

According to ASBMR guidelines for atypical fractures the following pharmacological treatment is recommended: discontinuation of BPs, continual supply of calcium and vitamin D. Teriparatide administration should be considered in cases of a lack of normal fracture healing (recommendations without any randomized, placebo-controlled trials) [27]. In our case we did not change the prior treatment scheme because the fracture union was normal. Moreover, converting on Teriparatide may not protect the contralateral femoral neck from fracture as well [14].

In conclusion, the presented fracture is caused by normal loading of an abnormal, osteoporotic bone and intake of BPs (the insufficiency fracture associated with BPs treatment). It is important to note there are always possibilities of new variant fractures, which are a combination of fatigue and atypical fractures [11]. We strongly recommend that every patient undergoing treatment using BPs should be warned about the risk of non- traumatic fractures, even in the femoral neck. The patient should be advised to report pain immediately, adequate diagnostic tests must be performed, and a treatment plan must be implemented as soon as possible. Therapy using BPs should be reviewed on a regular basis. There is a relatively low risk of side effects associated with BP usage, but nevertheless it must be compared to non-trivial consequences induced by lack of such treatment [27, 28].
